# Dose rate response of Digital Megavolt Imager detector for flattening filter‐free beams

**DOI:** 10.1002/acm2.12358

**Published:** 2018-05-21

**Authors:** Zhigang Xu, Jinkoo Kim, James Han, An Ting Hsia, Samuel Ryu

**Affiliations:** ^1^ Department of Radiation Oncology Stony Brook University Hospital Stony Brook NY USA

**Keywords:** FFF, PDC, IMRT, VMAT, EPID, QA

## Abstract

In this study we investigated the dose rate response characteristics of the Digital Megavolt Imager (DMI) detector, including panel saturation, linearity, and imager ghosting effects for flattening filter‐free (FFF) beams. The DMI detector dose rate response characteristics were measured as a function of dose rate on a Varian TrueBeam machine. Images were acquired at dose rates ranging from 400 to 1400 MU/min for 6XFFF and 400 to 2400 MU/min for 10XFFF. Line profiles and central portal doses derived from the images were analyzed and compared. The linearity was verified by acquiring images with incremental Monitor Unit (MU) ranging from 5 to 500 MU. Ghosting effects were studied at different dose rates. Finally, for validation, test plans with optimal fluence were created and measured with different dose rates. All test plans were analyzed with a Gamma criteria of 3%‐3 mm and 10% dose threshold. Our study showed that there was no panel saturation observed from the profile comparison even at the maximum dose rate of 2400 MU/min. The central portal doses showed a slight decrease (1.013–1.008 cGy/MU for 6XFFF, and 1.020–1.009 cGy/MU for 10XFFF) when dose rate increased (400–1400 MU/min for 6XFFF, and 400–2400 MU/min for 10XFFF). The linearity of the DMI detector response was better than 0.5% in the range of 20–500 MU for all energies. The residual image was extremely small and statistically undetectable. The Gamma index measured with the test plans decreased from 100% to 97.8% for 6XFFF when dose rate increased from 400 to 1400 MU/min. For 10XFFF, the Gamma index decreased from 99.9% to 91.5% when dose rate increased from 400 to 2400 MU/min. We concluded that the Portal Dosimetry system for the TrueBeam using DMI detector can be reliably used for IMRT and VMAT QA for FFF energies.

## 1 INTRODUCTION

The Electronic Portal Imaging Devices (EPID) is a very useful device for routine clinical use because of its prompt setup, easy data acquisition, and high resolution. The Portal Dosimetry is an application integrated on the ARIA electronic medical record system (Varian Medical Systems VMS, Palo Alto, CA, USA) that allows IMRT or VMAT field verification. The dosimetry process consists of three steps: (a) Fluence prediction using Eclipse Portal Dose Calculation (PDC) algorithm, (b) Fluence acquisition using dosimetry mode on the linac, and (c) Fluence comparison using the Portal Dosimetry module in Aria. The Portal Dosimetry adds a fast and efficient workflow for the verification of IMRT and VMAT plans.^1^

Since the option of removing the flattening filter (FF) in the linacs for IMRT and VMAT treatments was introduced in 2010, there has been a lot of interest generated in using flattening filter‐free (FFF) beams which give the benefit of reduced headscatter and hence, reduced dose outside the field.^2^ These beams also deliver dose faster than flattened beams, which would be beneficial for hypofractionated treatments by reducing treatment time and potential intrafractional organ motion.^3^

The Digital Megavolt Imager (DMI) detector is now a standard MV imaging detector installed on all Varian TrueBeam machines. The DMI detector offers not only the possibility to image large field size (43 × 43 cm^2^) but also the images with a higher pixel resolution (1280 × 1280) than the older detectors (i.e., IDU20, aS1000). The detector area used for dosimetry measurements (integrated images) is a little smaller than the complete imaging size (40 × 40 cm^2^ with 1190 × 1190 pixel).^4^ With faster readout electronics and a higher pixel capacitance, the DMI detector allows for much higher dose rate than the older detectors (i.e., IDU20, aS1000). It has been adapted by Varian for use in FFF beams at any source‐to‐detector distance.^5^

Since the dose rates for FFF beams are up to six times higher than for conventional flattened beams, portal images taken at maximum FFF dose rate may saturate the images. The EPID saturation occurs when no additional photocurrent outputs from the photodiode as the incident optical power increases. There are a number of parameters that can affect the saturation limit. However, the detailed information of the hardware and software components is proprietary and unknown to the public. Several studies investigated the feasibility of using Portal Dosimetry to FFF beams for IMRT and VMAT plan verification.^6^, ^7^, ^8^, ^9^, ^10^ Chuter et al. studied the Portal Dosimetry of Elekta Synergy linear accelerator (Elekta, AB, Stockholm, Sweden) with an Agility collimator capable of delivering FFF beams. They found that, with iViewGT amorphous silicon (aSi) panel (RID 1640 AL5P) at a fixed SSD of 160 cm, images taken at maximum FFF dose rate could saturate the EPID. A dose rate of 800 MU/min was found not to saturate the EPID for open fields.^6^ Pardo et al. presented a method for FFF Portal Dosimetry by placing the EPID at a greater distance to avoid panel saturation with Varian TrueBeam STX 1.6 and an aS1000 model.^7^ Miri et al. studied the Portal Dosimetry of a TrueBeam with an aS1200 panel for flattened filter (FF) and flattening filter‐free (FFF) beams. They studied the linearity of dose–response with MU, the imager lag, and the effectiveness of backscatter shielding. They concluded that significant improvements were observed in the dosimetric response of the aS1200 imager compared to previous imaging detectors (IDU20, aS1000).^5^

To the best of our knowledge, the study of the dose rate response characteristics for the DMI detector has not been reported in the literature. The purpose of this work was to study the panel saturation as a function of the dose rate, including dose linearity and imager ghosting effects of the DMI detector for FFF beams.

## 2 MATERIALS AND METHOD

### 2.A Portal dosimetry commissioning

The Portal Dosimetry was used to acquire delivered dose images, while Eclipse was used to compute a corresponding dose distribution. Both images were viewed and quantitatively compared in the Portal Dosimetry. The PDC was used to calculate portal dose images for fields containing fluences as part of a pre‐treatment verification for IMRT and VMAT planning.^11^

The portal imager was calibrated in dosimetric acquisition mode at isocenter according to the official Portal Dosimetry calibration procedure.^12^ The procedure includes applying a dark and flood‐field correction, an absolute calibration, and a beam profile correction. The absolute calibration is defined using 100 MU to correspond to 1 calibrated unit (CU) with a 10 × 10 cm^2^ field. The beam profile correction was made using diagonal profiles measured in a water phantom at a depth of maximum dose (dmax). During the commissioning of Portal Dosimetry, all calibration datasets were measured at 400 MU/min to eliminate potential panel saturation.

The configuration of the Eclipse PDC algorithm requires output factor measurements measured with the DMI panel, the beam intensity profile and a set of pyramid‐shaped test images from which the algorithm configuration derives the DMI pencil beam kernel.^12^ The pyramid‐shaped fluence provided by Varian is an optimal fluence specially designed for the configuration and verification of PDC. The fluence consists of a port of 120 × 250 mm with five rectangular slabs of the intensity of 1.0 and fluence of 0.0 outside the slabs.

**Figure 1:**
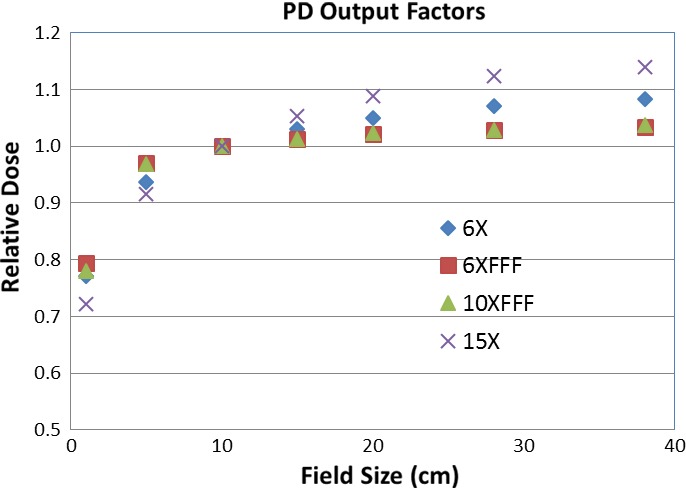
Output factors are plotted as a function of square field size.

Output factors were acquired with the imager panel at isocenter for field sizes ranging from 1 × 1 to 38 × 38 cm^2^. Figure 1 displays the output factors as a function of square field size for all the energies on our TrueBeam machine. The beam intensity profiles were derived from the previously acquired diagonal beam profiles at dmax in a water phantom for the configuration of the Eclipse AAA photon dose calculation algorithm.

**Figure 2:**
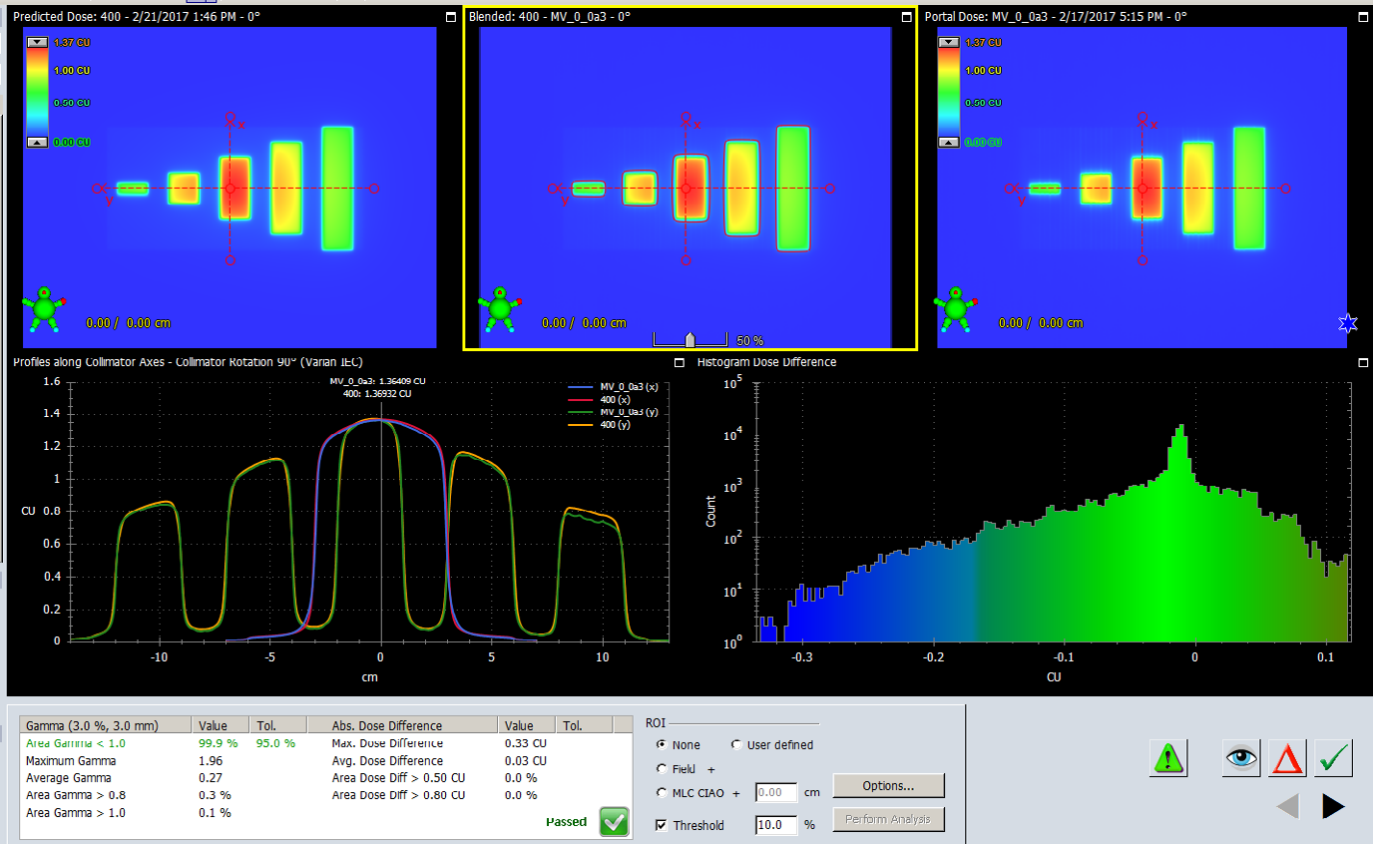
Portal Dosimetry user interface: Predicted Dose (up‐left), Evaluated Dose (up‐center), Portal Dose (up‐right), Profiles (bottom‐left), and Histogram (bottom‐right).

After the PDC was configured as described above, the Portal Dosimetry was tested with fields that have pyramid‐shaped fluence imported. The actual fluences were then recalculated using the Eclipse Smart Leaf Motion Calculation (LMC 13.6.23), and the portal dose images were subsequently predicted, measured, and analyzed for all energies. All of the measurements met the Varian acceptance criteria: Dose Difference: 4%, Distance‐To‐Agreement: 4 mm, Gamma pass rate: >99%. The Portal Dosimetry user interface is shown in Fig. 2.

### 2.B Profile and central point dose measurement as function of dose rate

The DMI detector dose rate response characteristics were studied as a function of dose rate. Images were acquired at dose rates from 400 to the maximum of 1400 and 2400 MU/min for 6XFFF and 10XFFF, respectively, in six steps, delivering a 100 MU 20 × 20 cm^2^ field. Line profiles and central portal doses derived from the images were compared and analyzed. The central portal doses were measured as the average of pixel value in the center of the field, using the output factor tool of the Portal Dosimetry.

### 2.C The linearity of the DMI detector response

The linearity of the DMI detector response as a function of MU was investigated by delivering a 10 × 10 cm^2^ field with MU ranging from 5 to 500 for both of the unflattened modes (6XFFF and 10XFFF) and the conventional 6X mode, for clinical dose rates at 1400, 2400, and 400 MU/min, respectively.

### 2.D The dose rate response of ghosting effects

**Figure 3:**
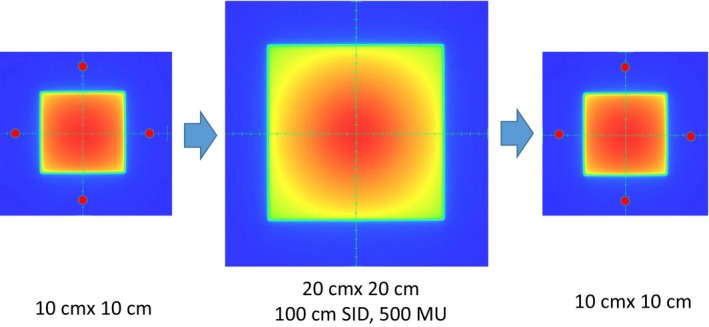
The residual image (ghost) was studied by measuring imager lag after 500 MU delivery in the periphery region (four points in red).

The dose rate response of ghosting effects was studied by measuring the imager lag after delivering 500 MU at different dose rates for all energies. A 10 × 10 cm^2^ treatment field was acquired immediately after exposing the DMI detector using a 20 × 20 cm^2^ field. The time delay between two consecutive images was approximately 30 s which is the time delay required by the TrueBeam machine. The residual image (ghost) in the periphery region outside the central 10 × 10 cm^2^, but within the 20 × 20 cm^2^ treatment field, was carefully checked by comparing the portal doses in the same region that was acquired after a long time interval without image acquisitions (see Fig. 3 for illustration). The residual image (RI) is then

(1) $$RI=\frac{PD_{\mathrm{After}}-PD_{\mathrm{Before}}}{PD_{\mathrm{Center}}}\times 100\%$$

where *PD*_*Before*_ and *PD*_*After*_ are the portal doses before and after 20 × 20 cm^2^ treatment field is delivered, respectively. *PD*_*Center*_ is the portal dose at filed center for 10 × 10 cm^2^ field size. An average of RI calculated based on four points at 8 cm away from the center was used to determine the residual signals.

Finally, plans with pyramid‐shaped fluence were created. Portal images of the field were acquired at 6XFFF and 10XFFF energies delivered at different dose rates. All test plans were analyzed with a gamma criteria of 3%‐3 mm and 10% dose threshold, which is a typical gamma criteria for IMRT QA in our institution.

## 3 RESULTS AND DISCUSSION

**Figure 4:**
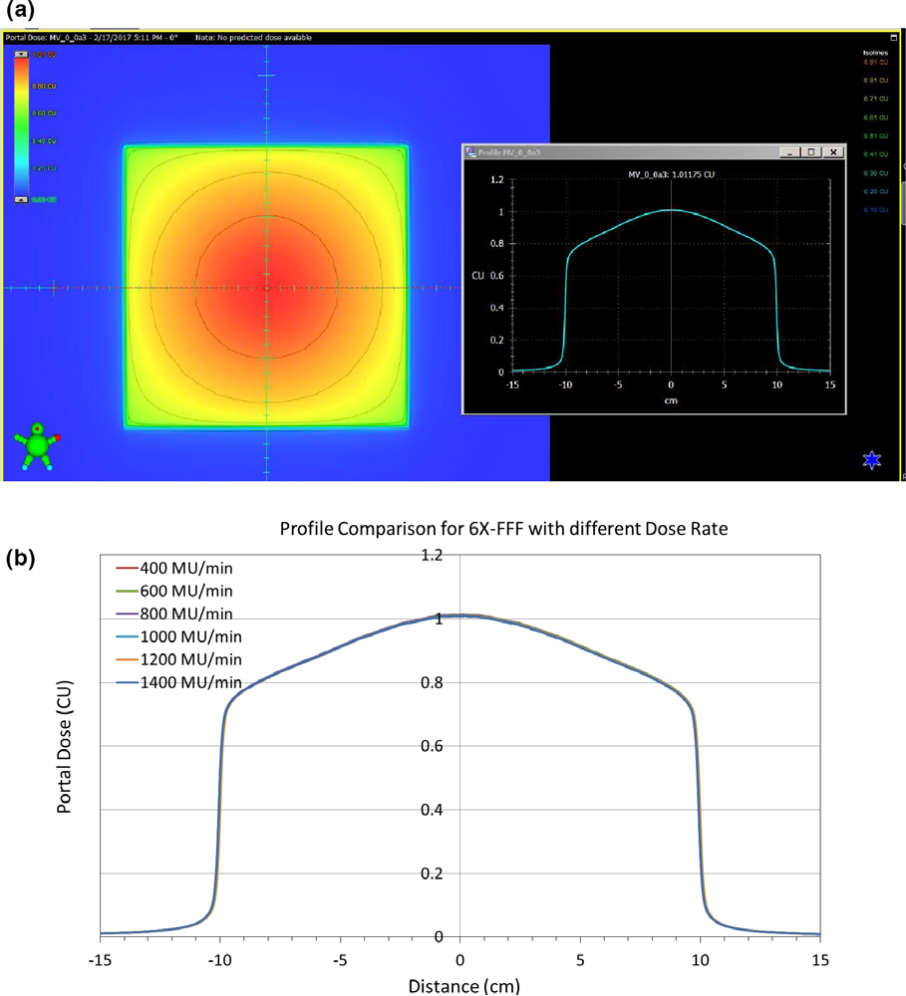
Portal dose image (a) and line profiles (b) at different dose rates for 6XFFF.

**Figure 5:**
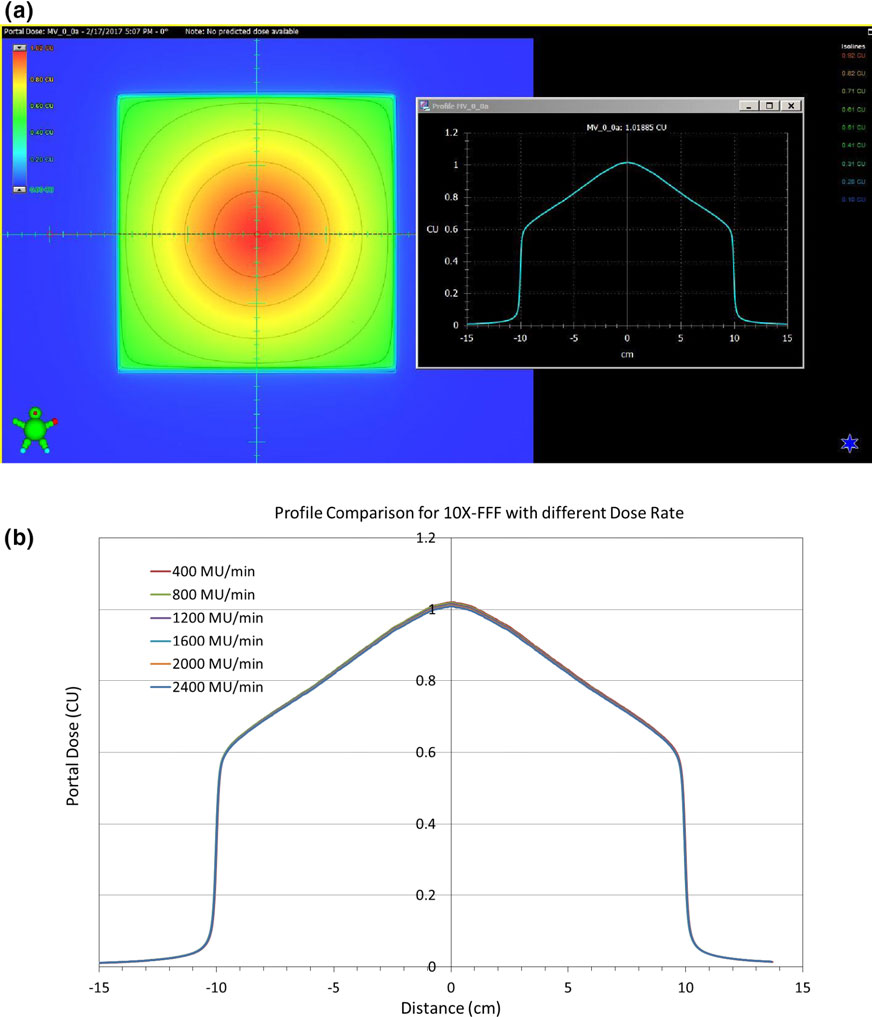
Portal dose image (a) and line profiles (b) at different dose rates for 10XFFF.

**Figure 6:**
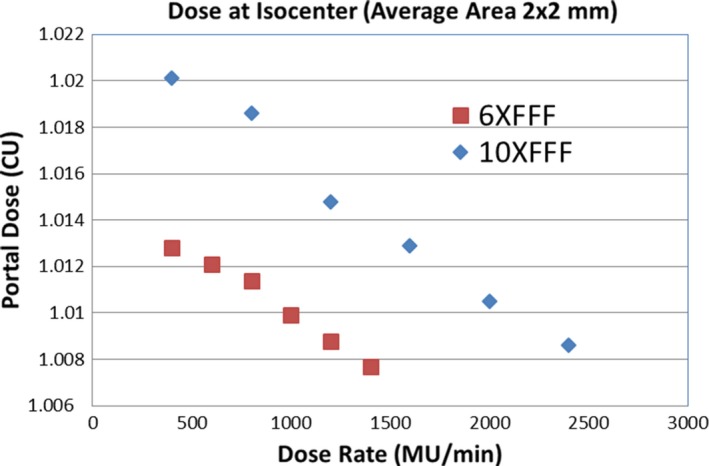
Central portal doses as a function of dose rate for 100 MU and 20 × 20 cm^2^ field size.

**Figure 7:**
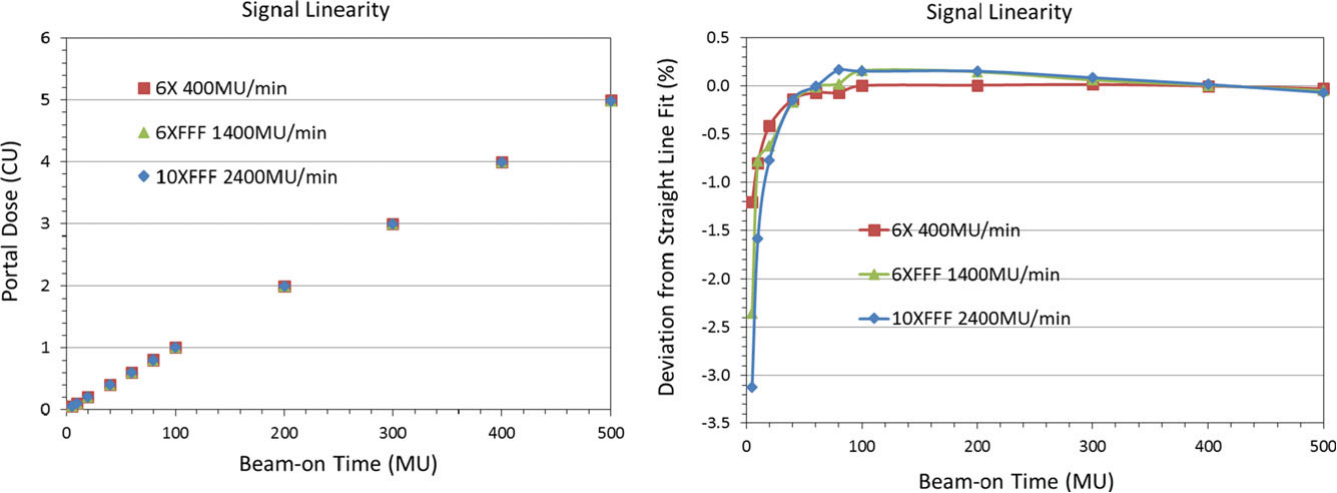
(a, b) Image response is linear in wide range of MU (20–500MU).

The portal dose image and their line profiles for 100 MU and 20 × 20 cm^2^ field size for 6XFFF at different dose rates are shown in Figs. 4(a) and 4(b), respectively. The corresponding image and line profiles of 10XFFF are shown in Figs. 5(a) and 5(b). No panel saturation was detected even with a maximum dose rate of 2400 MU/min for 10XFFF by comparing the profiles with different dose rates. Figure 6 displays the central portal dose as the function of dose rate for 100 MU and 20 × 20 cm^2^ field size for energies 6XFFF (in red) and 10XFFF (in blue). As mentioned above, the central portal dose was measured as the average of pixel value in the center of the field. This was done using the output factor tool of the Portal Dosimetry. A slight decrease in central portal dose, from 1.013 to 1.008 cGy/MU, was observed when the dose rate increased from 400 to 1400 MU/min for 6XFFF. A similar decrease, from 1.020 to 1.009 cGy/MU, was measured for 10XFFF when the dose rate increased from 400 to 2400 MU/min. However, both dose reductions are less than 1.09%, meaning the dose rate effect is clinically insignificant. Results of the linearity tests are shown in Fig. 7 (a) for the conventional flattened beam (6 MV) and unflatten beams (6XFFF and 10XFFF). All dose rates were calibrated to yield 1 CU per 100 MU. The linearity was better than 0.5% in wide range of MU (20–500 MU) for all energies as shown in Fig. 7 (b).

The measured residual image signals at different dose rates for 6XFFF and 10XFFF were extremely small (<0.1%) and statistically undetectable after delivering 500 MU. Finally, the results of the Gamma analysis (3%‐3 mm) for test plans with different dose rates are summarized in Table 1. The Gamma agreement index was decreased from 100% to 97.8% when dose rate increased from 400 to 1400 MU/min for 6XFFF, and from 99.9% to 91.5% when dose rate increased from 400 to 2400 MU/min for 10XFFF. As the data shown in Fig. 6 for 10XFFF, only a slight decrease (about 1%) of central portal dose was observed for an open field when the dose rate increased from 400 to 2400 MU/min. The decrease in Gamma agreement index here is mainly due to the dose rate response of the Multileaf Collimator (MLC) motion. Increasing the dose rate increases the number of control points per unit time and, thus, it increases the complexity of the MLC delivery. Lowering the dose rate helps to improve the gamma pass rate. This is in agreement with the work done by Kaviarasu et al.^13^

**Table 1:** Gamma analysis for test plans with different dose rates (3%‐3 mm)

Dose rate (MU/min)	6XFFF	Dose rate (MU/min)	10XFFF
400	100	400	99.9
600	99.9	800	99.8
800	99.8	1200	99.2
1000	99.7	1600	97.5
1200	98.8	2000	95.5
1400	97.8	2400	91.5

**Figure 8:**
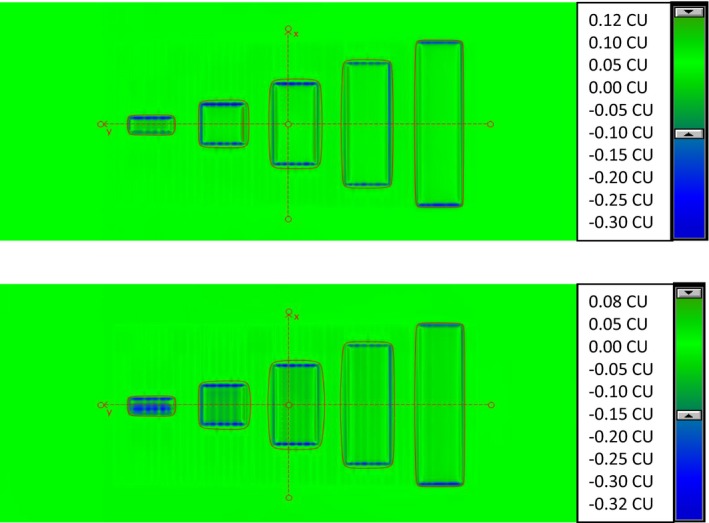
Portal dose difference images for 10XFFF: 400 MU/min dose rate (top) and 2400 MU/min dose rate (bottom).

Figure 8 shows the portal dose difference images (measured image–predicated image) for 10XFFF with dose rate of 400 MU/min (top) and 2400 MU/min (bottom). The highest dose difference was appeared near the 1–2 cm bands with a bigger difference for 2400 MU/min dose rate beam. With the consideration of the extreme dose gradients of the high‐dose rate beams, the low passing rate near the short band width is clinically acceptable. In general, the fluence patterns of clinical plans will have smoother profiles and the machine is unlikely to be running at a constant maximum dose rate of 2400 MU/min.

## 4 CONCLUSION

The Portal Dosimetry utilizing EPID is an existing technique that has been shown to work well with standard, flattened radiotherapy beams. With the emerging use of high‐dose‐rate FFF radiotherapy there is an associated need to verify these treatments efficiently. This study has shown that the DMI panel saturation was clinically insignificant even at the maximum dose rate of 2400 MU/min. The linearity of the DMI detector response was better than 0.5% in the range of 20–500 MU for all energies. The residual image (ghost) was extremely small and statistically undetectable. With faster readout electronics and a higher pixel capacitance, the DMI detector allows for a much higher dose rate than the older detectors (i.e., IDU20, aS1000) without saturation. Therefore, we conclude that the Portal Dosimetry system for the TrueBeam with the DMI detector can be reliably used for IMRT and VMAT pretreatment QA verification for FFF energies.

## CONFLICTS OF INTEREST

The authors do not have any conflicts of interest to declare.
